# Tumor-Educated Platelet miR-18a-3p as a Novel Liquid-Biopsy Biomarker for Early Diagnosis and Chemotherapy Efficacy Monitoring in Nasopharyngeal Carcinoma

**DOI:** 10.3389/fonc.2021.736412

**Published:** 2021-10-06

**Authors:** Kaiyu Sun, Hui Wang, Xianqun Xu, Xiuqi Wei, Jingyu Su, Kaidong Zhu, Junli Fan

**Affiliations:** ^1^ Department of Otolaryngology, Zhongnan Hospital of Wuhan University, Wuhan, China; ^2^ Department of Laboratory Medicine, Union Hospital, Tongji Medical College, Huazhong University of Science and Technology, Wuhan, China; ^3^ Department of Laboratory Medicine, Zhongnan Hospital of Wuhan University, Wuhan, China; ^4^ Department of Laboratory Medicine, Cancer Center, Union Hospital, Tongji Medical College, Huazhong University of Science and Technology, Wuhan, China

**Keywords:** tumor-educated platelet, miR-18a-3p, liquid-biopsy, early diagnosis, chemotherapy, nasopharyngeal carcinoma

## Abstract

**Aims:**

To evaluate the value of tumor-educated platelet (TEP) miR-18a-3p in the early diagnosis and chemotherapy efficacy monitoring of nasopharyngeal carcinoma (NPC).

**Methods:**

Expression levels of miR-18a-3p in platelets and plasma were detected by relative quantitative real-time PCR in NPC patients (n=54) and normal subjects (n=36). Diagnostic values of TEP miR-18a-3p for NPC was assessed by receiver operating characteristic (ROC) curve analysis. Follow up study was carried out to observe the dynamic changes of TEP miR-18a-3p with chemotherapy on 3 NPC patients.

**Results:**

The expression levels of TEP miR-18a-3p in NPC patients were significantly higher than that in healthy controls (*p* < 0.0001). ROC curve analysis showed that the area under the curve (AUC) value was 0.841, the sensitivity and specificity for the diagnosis of NPC were 87% and 72.7%. No correlation was found between expression levels of TEP miR-18a-3p and patients’ clinical parameters and their NPC tumor-node-metastasis (TNM) stage. The positive rate of TEP miR-18a-3p and EBV DNA for NPC diagnosis were 85.4% and 66.7%. TEP miR-18a-3p expression were down-regulated after 77.8% (7 of 9) of chemotherapy, and in 66.7% (2 of 3) patients, TEP miR-18a-3p levels decreased after 3 cycles of chemotherapy.

**Conclusion:**

The expression levels of TEP miR-18a-3p are upregulated in NPC and have a high probability to downregulated after chemotherapy, indicating a significant clinical value. TEP miR-18a-3p might serve as a novel type of liquid-biopsy biomarker for early diagnosis and chemotherapy efficacy monitoring in NPC.

## Introduction

Nasopharyngeal carcinoma (NPC) is a common malignancy arising in the nasopharynx epithelium with a remarkable ethnic and geographic distribution. NPC has a particularly high prevalence in eastern and southeastern Asia and southern China (1–3). The pathogenesis of NPC is complex and diverse, the etiology of NPC includes genetic susceptibility, dietary habits and Epstein-Barr virus (EBV) infection (4). Early symptoms of NPC include headache, neuro facial pain, neck mass, nosebleed or stuffy nose, NPC may easily be misdiagnosed since the early symptoms are non-specific (5). Radiotherapy and chemotherapy remains the mainstay of primary treatment for NPC. Non-keratinized, poorly differentiated and undifferentiated histological subtypes are particularly sensitive to chemotherapy, while radiotherapy can cure more than 90% of patients in stage I, but patients in advanced stages have a low 5-year survival rate due to local recurrence and distant metastasis (6). Therefore, early diagnosis of NPC is very important and necessary. Nasal endoscopic biopsy is the most commonly used method for diagnosis of NPC, but it is difficult to obtain tissue samples in early stage of the disease. Thus, there is an urgent need for the exploration of new biomarkers for non-invasive early detection.

MiRNAs are endogenous, small, non-coding RNA of 19 to 25 nucleotides that regulates gene expression by binding to the 3 untranslated region of target gene mRNA (7), have been demonstrated to regulate the development of many cancers by acting as tumor suppressor or oncogene in previous studies (8). MiRNAs have remarkable stability in solid and liquid specimens, so they can serve as a potential non-invasive biomarker (9), and offer a non-invasive opportunity for early diagnosis of cancer. Abnormal expression of miRNAs have been reported in the occurrence and development of NPC, such as MiR-34c, MiR124, miR-140-3p, miR-144-3p, miR-17-5p, miR-20a-5p, miR-20b-5p, and miR-205-5p (10–12). A recent study found that miR-18a was up-regulated in NPC samples, playing a role in the occurrence and development of nasopharyngeal carcinoma, and antagomir-18a might eventually be used in the treatment of NPC (13). Another study reported that miR-18a could promote proliferation of nasopharyngeal carcinoma cells by targeting miRNA processing enzyme DICER112 (14). However, the expression pattern of miR-18a-3p has not been investigated in nasopharyngeal carcinoma.

Platelets are multi-functional cell fragments best known as the cellular mediator of thrombosis. Platelets assist in sensing pathogens entering the blood stream, signaling to immune cells, releasing vascular remodeling factors, and negatively enhancing cancer metastasis. Platelets also contain a rich repertoire of RNA species, which is related to tumor biology and metastasis (15). The components in platelets can be affected by the specialized surrounding microenvironment, including tumor cells. Tumor cells appear to initiate intraplatelet signaling, resulting in splicing of platelet pre-mRNAs and enhance secretion of cytokines (15). In addition, platelet RNA appears to be dynamically affected by pathological conditions (15). It has been demonstrated that tumor cells influence platelet RNA through different mechanisms, resulting in tumor-specific RNA profiles (16). Tumor-educated platelet (TEP) RNA is involved in the progression and spread of several solid tumors, including lung, brain and breast cancer. Therefore, TEP RNA may serve as a liquid-biopsy biomarker in early diagnosis and monitoring treatment efficacy of cancer (17).

In this study, we investigated the expression levels of TEP miR-18a-3p in NPC patients and aimed to explore the value of TEP miR-18a-3p in early diagnosis and chemotherapy efficacy monitoring for NPC.

## Materials and Methods

### Study Design

All samples were collected at Cancer Center, Union Hospital, Tongji Medical College, Huazhong University of Science and Technology and Zhongnan Hospital of Wuhan University from 2017 to 2019. The study was approved by the Ethics Committee of Wuhan Union Hospital, Tongji Medical College, Huazhong University of Science and Technology and the Medical Ethical Committee of Zhongnan Hospital of Wuhan University. Written informed consent was obtained from all participants. 57 NPC patients and 36 healthy donors participated in this study. 54 patients had one sample taken before any treatment to assess the diagnostic value of TEP miR-18a-3p for NPC. For the remaining 3 patients, samples were collected before and after each dose of chemotherapy to monitor dynamic change of TEP miR-18a-3p during treatment. All patients were pathologically confirmed NPC cases. The clinical and histopathological features of each participant, such as sex, age, TNM stage, history of EBV infection and pathological type, were recorded in detail.

### Sample Collection

Whole blood samples (3 mL) were taken from each participant and collected into test tubes containing EDTA-K_2_. The blood samples were centrifuged at 120 g for 20 minutes at room temperature and the upper layer rich in platelets (about 900 μL) were collected. The platelets were precipitated by centrifugation at 360 g for 20min at room temperature, the supernatant was plasma. Pipetting 300 μL of plasma from the supernatant into a new centrifuge tube, then discarding the remaining plasma, the precipitates were platelets. The platelets were washed twice by phosphate buffer saline. A high purity platelet preparation was determined by a ratio of < 5 karyocytes per 10^7^ platelets (18). After centrifugation, platelets and plasma were added with 500 μL RNA lysate (a reagent that lysed cells and inactivated RNase included in a Liquid Total RNA Isolation Kit) and mixed for 5 minutes, respectively, then stored in -80°C refrigerator ready for RNA extraction and amplification.

### RNA Extraction and Relative Real-Time Quantitative PCR (qPCR) Analysis

The total RNA was extracted from platelets or plasma using a Liquid Total RNA Isolation Kit (RP4002, BioTeke, Beijing, China). The quantity and quality of RNAs were determined by Nanodrop 2000 spectrophotometer and analyzed by an agarose gel electrophoresis. In total, 1 μg of RNAs were used for cDNA synthesis using a PrimeScript™ RT reagent Kit with gDNA Eraser (RR047A, TAKARA, Dalian, China). The qPCR was performed using iQ™ SYBR^®^ Green Supermix (BIO-RAD, CA, USA) on a BIO-RAD CFX96 system. The U6 was used as the internal amplification control. The primers used for amplification of miR-18a-3p and U6 are listed in **Table 1**. The URP was universal amplification primer, which was used in pairs with miR-18a-3p-S. The relative expression levels of miRNAs were calculated by 2^−△CT^ method; while △CT = CT_miR18a-3p_ - CT_U6_. EBV DNA levels were determined using EB viral nucleic acid quantitative fluorescent probe PCR assay (Shengxiang Biotechnology, Hunan, China) on the Stratagene Mx3000P system (Agilent Technologies, CA, USA) at Department of Laboratory Medicine of Union Hospital.

**Table 1:** Primers used for cDNA synthesis and real-time PCR.

Primers	Sequence (5’~3’)
miR-18a-3p-S	ACACTCCAGCTGGGACTGCCCTAAGTGCTCC
miR-18a-3p-RT*	CTCAACTGGTGTCGTGGAGTCGGCAATTCAGTTGAGCCAGAAGG
URP	TGGTGTCGTGGAGTCG
U6-F	CTCGCTTCGGCAGCACA
U6-RT*	AACGCTTCACGAATTTGCGT

*cDNA synthesis primers.

### Data Analysis

SPSS21.0 software was used for statistical analysis. MedCalc statistical software was used for ROC analysis. Mann-Whitney U test was used to compare the expression of miR-18a-3p in NPC and healthy donors, and subtypes of different TNM stages. The expression levels of miR-18a-3p were showed as (median, (lower quartiles, upper quartiles)). Independent sample t-test and Fisher’ exact test were used to determine the correlation between the expression level of miR-18a-3p and the clinical parameters of the subjects. The positive rates of miR-18a-3p and EBV DNA in the diagnosis of NPC were compared by McNemar and Kappa test. A two-sided test was performed in all statistical analyses. Statistically, significant difference was set at *p* < 0.05.

## Result

### Relative Expression Levels of miR-18a-3p in Platelet and Plasma of NPC Patients and Healthy Controls

**Figure 1:**
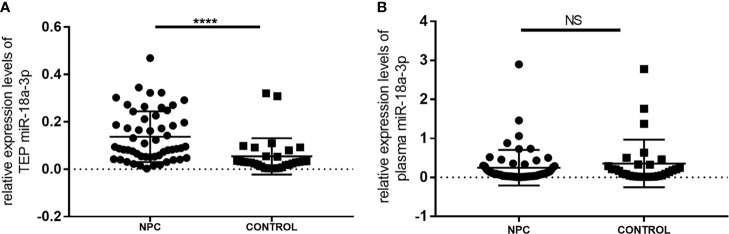
Relative expression levels of miR-18a-3p in platelet and plasma of NPC patients and healthy controls. **(A)** The expression levels of TEP miR-18a-3p in NPC patients were significantly higher than that in healthy controls. **(B)** There was no significant difference of plasma miR-18a-3p levels between NPC and control groups. Mann–Whitney U test was used for analysis. ****p < 0.0001. NS, no significance.

The expression levels of TEP miR-18a-3p in NPC patients [0.0914 (0.0519, 0.2155)] were significantly higher than that in healthy controls [0.0300 (0.0162, 0.0607)] (*p* < 0.0001, **Figure 1**). However, there was no significant difference in the expression levels of miR-18a-3p in plasma between the two groups (*p* = 0.368, **Figure 1**).

### Evaluation of the Diagnostic Value of miR-18a-3p in NPC

**Figure 2:**
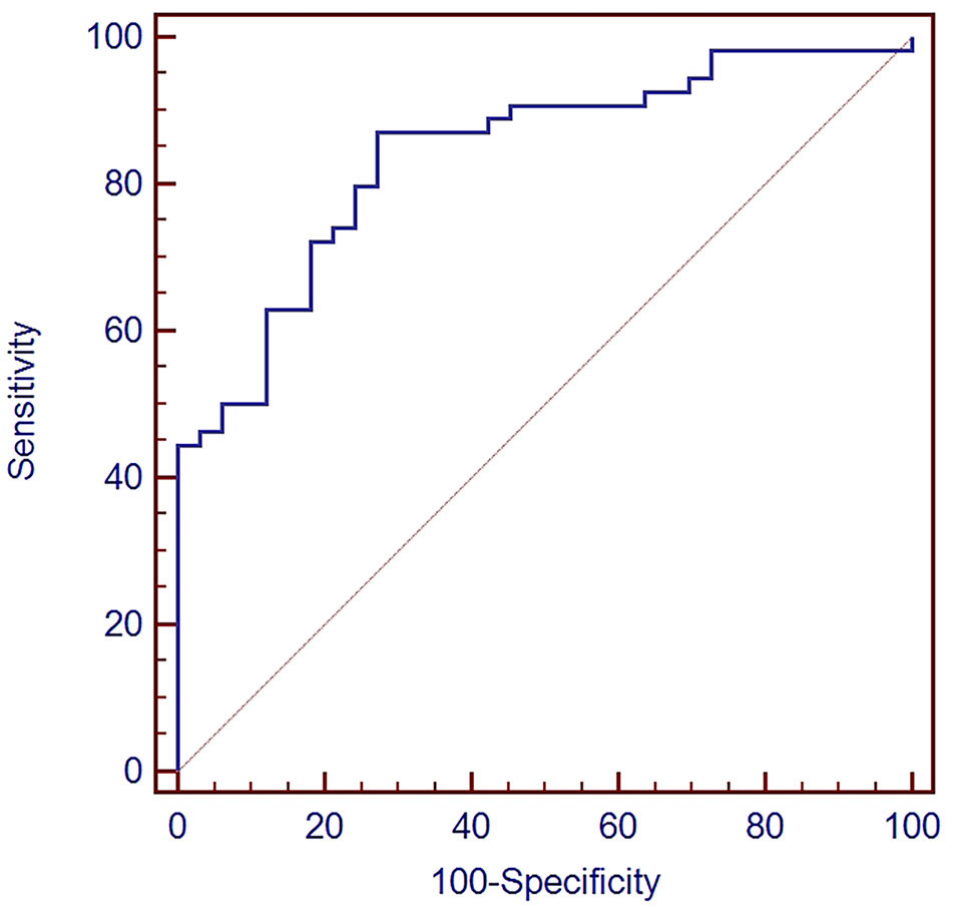
ROC of miR-18a-3p in TEP for NPC.

ROC curve analysis was used to evaluate the diagnostic value of TEP miR-18a-3p in NPC. The area under the curve (AUC) value was 0.841 (95% CI: 0.747~0.910), Youden index was 0.5976 and the criterion was > 0.0357. The sensitivity and specificity for the diagnosis of NPC were 87% and 72.7%, respectively (**Figure 2**).

### Correlations Between the Expression Levels of TEP miR-18a-3p and the Clinical Parameters of NPC

**Figure 3:**
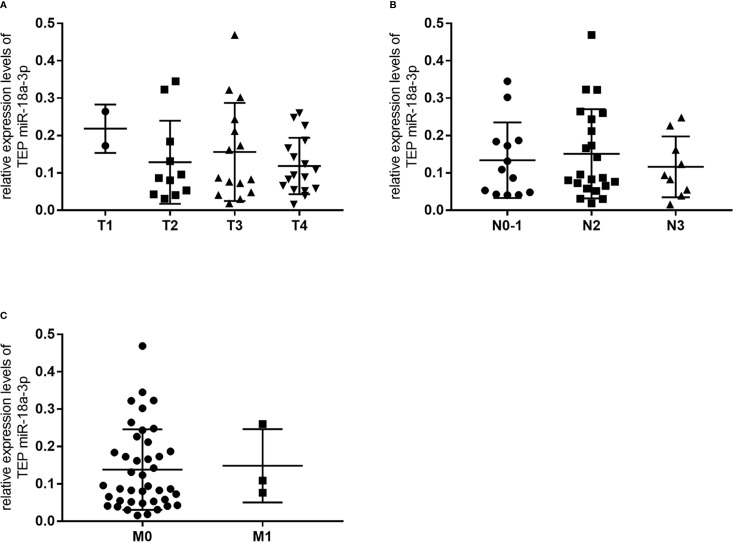
There was no significant change in relative expression levels of TEP miR-18a-3p among different TNM stages of NPC. **(A)** Relative expression levels of TEP miR-18a-3p in T1-T4 stages; **(B)** Relative expression levels of TEP miR-18a-3p in N0~1, N2, and N3 stages; **(C)** Relative expression levels of TEP miR-18a-3p in M0 and M1 stages. Mann–Whitney U test was used for analysis.

We further investigated the relationship between TEP miR-18a-3p and NPC patients’ clinical parameters and their TNM stages. NPC patients were divided into high-expression group and low-expression group according to the cut-off point (0.0357) of miR-18a-3p in the Youden index analysis. The results showed that 87% (47 of 54) of the patients had high miR-18a-3p expression, but there was no significant difference in age (*p*=0.392, **Table 2**), gender (*p*=0.226, **Table 2**), hypertension (*p*=0.576, **Table 2**), diabetes (*p*>0.999, **Table 2**), Nasopharyngitis (*p*=0.322, **Table 2**), smoking (*p*>0.999, **Table 2**), alcohol drinking (*p*>0.999, **Table 2**), degree of differentiation (*p*=0.436, **Table 2**), TNM stage (*p*=0.806, 0.416, 0.58, respectively, **Table 2**) and EBV-DNA copies (*p*=0.392, **Table 2**). However, the positive rate of miR-18a-3p (85.4%) was higher than that of EBV-DNA (66.7%). Statistical analysis showed that there was no significant difference in the expression levels of miR-18a-3p among different TNM stages of NPC (*p*>0.05, **Figure 3** and **Table 3**).

**Table 2:** Correlations between expression levels of TEP miR-18a-3p and clinical parameters of NPC.

Clinical parameters	N	TEP miR-18a-3p			*p*-value
		Low	High	High%	
Age	54	45.85 ± 9.83	49.55 ± 10.67	–	0.392^b^
		n=7	n=47	
Gender					
Male	35	3	32	91.4	0.226
Female	19	4	15	78.9
Hypertension	8	0	8	100	0.576
Diabetes	6	1	5	83.3	>0.999
Nasopharyngitis	11	0	11	100	0.322
Smoking	12	1	11	91.7	>0.999
Alcohol drinking	6	0	6	100	>0.999
Tumor type					
Undifferentiated	50	6	44	88.0	0.436
Differentiated	4	1	3	75.0
T classification					
T1	2	0	2	100.0	0.806
T2	11	1	10	90.0
T3	15	2	13	86.7
T4	17	1	16	94.1
Lymph node status					
N0~N1	12	0	12	100.0	0.416
N2	22	3	19	86.4
N3	9	1	8	88.9
Metastatic status					
M0	43	4	39	90.7	0.580
M1	3	0	3	100.0
EBV DNA					
Negative	16	2	14	87.5	0.064^a^
Positive	32	5	27	84.4

Positive rate of TEP miR-18a-3p: 85.4%.

Positive rate of EBV DNA: 66.7%.

^a^McNemar and Kappa tests, ^b^independent-samples t-test, all others are Fisher test.

**Table 3:** The *p* values of comparing relative expression levels of TEP miR-18a-3p among groups with different TNM stages of NPC.

	T1-T2	T1-T3	T1-T4	T2-T3	T2-T4	T3-T4	N0~1-N2	N0~1-N3	N2-N3	M0-M1
miR-18a-3p	0.31	0.44	0.11	0.84	0.82	0.74	0.77	0.74	0.56	0.66

### Clinical Value of TEP miR-18a-3p in Monitoring the Efficacy of Chemotherapy for NPC

**Figure 4:**
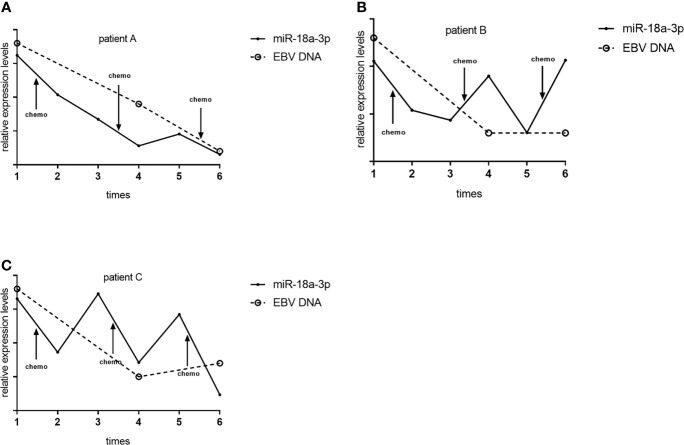
Dynamic changes of TEP miR-18a-3p and EBV DNA with chemotherapy. **(A)** Dynamic changes of TEP miR-18a-3p and EBV DNA with chemotherapy of patient **(A, B)** Dynamic changes of TEP miR-18a-3p and EBV DNA with chemotherapy of patient **(B, C)** Dynamic changes of TEP miR-18a-3p and EBV DNA with chemotherapy of patient **(C)** chemo, chemotherapy.

We followed up and investigated the levels of TEP miR-18a-3p of 3 NPC patients. Each patients accepted 3 times of chemotherapy, and the chemotherapy schemes of 3 patients were paclitaxel combined with cisplatin, gemcitabine combined with cisplatin, gemcitabine combined with cisplatin, respectively. TEP miR-18a-3p levels were detected before and after every chemotherapy, respectively. EBV DNA copies were obtained from the electronic medical record system according to the corresponding time. TEP miR-18a-3p expression were down-regulated after 77.8% (7 of 9) of chemotherapy, in 66.7% (2 of 3) of patients, TEP miR-18a-3p levels decreased after 3 cycles of chemotherapy. (**Figure 4**). Simultaneously, EBV DNA copies decreased after 83.3% (5 of 6) of chemotherapy, and 100% of the patents’ EBV DNA copies were decreased after 3 cycles of chemotherapy (**Figure 4**). The dynamic changes of TEP miR-18a-3p and EBV DNA with chemotherapy were roughly the same.

## Discussion

MicroRNAs are short non-coding single-stranded RNA that regulates gene expression by binding to complementary sequences in 3 or 5 untranslated region of target gene mRNA. Some studies have reported that MicroRNAs may play an important role in the pathogenesis of various human cancers (19). MicroRNAs can induce abnormal metabolism of tumor cells by affecting the process of transcription and translation of tumor cells. High expression of MiRNAs can lead to abnormal proliferation and migration of tumor cells and induce cancer, but in other cases, the role of miRNAs may be tumor inhibition (20). MicroRNA expression profiles show that they are related to development, progression and response of tumor to therapy, suggesting that they have a great potential to be considered as diagnostic, predictive and prognostic biomarkers of tumor (7, 8).

Nasal endoscopy and clinical imaging examination are the main methods to diagnose NPC. Biopsy samples are obtained for pathological diagnosis, when no tumor is visible and the suspect index is high, blind or radiology-guided targeted biopsy will be performed. However, benign condition-like tuberculosis of the nasopharynx can also present with a similar range of symptoms, In addition, it is difficult to detect distant metastasis by computed tomography (CT) and magnetic resonance imaging (MRI) at early stage, especially when the diameter of the lesion is less than 5 mm, which will delay the detection of the tumor. At the same time, PET-CT/CT are unaffordable for most patients, especially in underdeveloped and developing countries. Nasopharyngeal tissue biopsy is the gold standard for the diagnosis of NPC, but it is invasive, which may cause some unanticipated complications in the patients at high risk, or it can only be only be effective in the middle to late stages of NPC. At this time, the effect of related treatment is not good enough (1, 21). Radiotherapy and chemotherapy remains the mainstay of primary treatment for NPC, but there are not many biomarkers used for treatment efficacy monitoring. Plasma EBV DNA is a commonly used biomarker, however, it is not applicable to NPC patients without EBV infection. Therefore, it is of great significance to find effective, non-invasive biomarkers for early diagnosis and chemotherapy efficacy monitoring in NPC.

Circulating tumor cells (CTCs), cell-free nucleotides, exosomes and TEPs are different sources of liquid biopsy for tumor-associated biomarkers and they play different roles in clinical application (22). Platelets are implicated in tumor biology and metastasis and can be changed on RNA and protein content by tumor cells (16, 18, 23, 24). Platelets contain a rich repertoire of RNA species and more than 500 different miRNAs have been detected in human platelets (25). Besides releasing factors, platelets can also sequester RNAs and proteins released by cancer cells. Thus, platelets actively respond to queues from local and systemic conditions, thereby altering their transcriptome and molecular content (15).We screened several RNAs that have been reported to be altered in NPC and found that the expression levels of TEP miR-18a-3p in NPC patients were significantly higher than that in normal subjects, but there were no significant difference in plasma miR-18a-3p expression levels. These data suggest that platelets may interact with tumor cells directly *via* different receptors or indirectly *via* different signaling molecules and lead to platelets RNA content changes. On the other hand, platelets may absorb and enrich miR-18a-3p released by tumor cells, therefore no difference was found between NPC patients and healthy donors in plasma. In addition, we followed-up 5 NPC patients (2 of the patients were excluded) and found that TEP miR-18a-3p expression were down-regulated after 77.8% of chemotherapy, in 66.7% of patients, TEP miR-18a-3p levels decreased after 3 cycles of chemotherapy. The chemotherapy schemes included paclitaxel combined with cisplatin, gemcitabine combined with cisplatin. Suzuki reported that inhibition of miR-18a-3p reduced proliferation of mesothelioma cells and sensitizes them to cisplatin (19), Rodriguez-Aguayo reported that miR-18a-3p was related to chemotherapy-resistant ovarian cancer (26). Their findings are similar to our study and support our conclusions. Plasma load of EBV DNA has been shown to be of prognostic value (27). EBV DNA copies decreased after 83.3% of chemotherapy and all the patents’ EBV DNA copies decreased after 3 cycles of chemotherapy, indicated that the treatments were effective. The dynamic changes of TEP miR-18a-3p are almost synchronized with EBV DNA copies, demonstrating the potential of TEP miR-18a-3p as an indicator of chemotherapy efficacy monitoring in NPC.

We used RT-qPCR to detect expression levels of TEP miR-18a-3p. Previous studies have demonstrated that none of the commonly used reference genes for normalization are universal for all tissue types or experimental situations (28–30). Platelet RNAs have a small amount and no reliable endogenous control miRNA has been identified in studying platelets miRNAs, U6 RNA is still the most common choice. We determined quantity and quality of RNAs and guaranteed 1 ug of RNAs were used for reverse transcription. To make the results reliable, the U6 RNA was set up as internal reference gene, and Ct value of U6 RNAs were differ within 1. TEP miR-18a-3p has certain sensitivity, specificity and accuracy in the diagnosis of NPC, and can reflect whether the chemotherapy is effective in a certain extent. These results indicate that TEP miR-18a-3p is a sensitive novel liquid-biopsy biomarker for early diagnosis and chemotherapy efficacy monitoring in NPC. However, TEP miR-18a-3p shows no difference in each TNM stage and other clinical parameters, this may be due to the small sample size, and the results can only be preliminary at this stage. Platelets are rich in peripheral blood and easy to obtained, detecting TEP RNA is convenience, economical, and non-invasive to patients. In addition, platelets have no nucleus so detecting platelets RNA can reduce interference from their own genome. Our assay is also easily applicable to clinical and automation applications since RT-qPCR is a very mature detection technology. Of course, this study has some limitations. The sample size for dynamic monitoring is small, and we only monitored the efficacy of chemotherapy, the efficacy of radiotherapy was not evaluated. These work will be completed in our follow-up research. A diagnostic consisting of more than a single miRNA might strengthen the overall approach. In general, this study found that TEP miR-18a-3p were upregulated in NPC and had a high probability to downregulated after chemotherapy. Our data suggest that TEP miR-18a-3p can potentially serve as an essential, non-invasive biomarker for early diagnosis and chemotherapy efficacy monitoring in NPC.

## Data Availability Statement

The raw data supporting the conclusions of this article will be made available by the authors, without undue reservation.

## Ethics Statement

The studies involving human participants were reviewed and approved by The Ethics Committee of Wuhan Union Hospital, Tongji Medical College, Huazhong University of Science and Technology The Medical Ethical Committee of Zhongnan Hospital of Wuhan University. The patients/participants provided their written informed consent to participate in this study.

## Author Contributions

Conception and design: JF, KS, and HW. Development and methodology: KS, HW, XX, XW, JS, and KZ. Acquisition of data: KS, HW, and XX. Analysis and interpretation of data: KS, HW, and XW. Preparation of the paper: JF, KS, and HW.

Study supervision: JF and HW. All authors contributed to the article and approved the submitted version.

## Funding

This work was supported by Health commission of Hubei Province scientific research project (No. WJ2019H042) to JF and Health Commission of Hubei Province scientific research project (No. WJ2019M158) to HW.

## Conflict of Interest

The authors declare that the research was conducted in the absence of any commercial or financial relationships that could be construed as a potential conflict of interest.

## Publisher’s Note

All claims expressed in this article are solely those of the authors and do not necessarily represent those of their affiliated organizations, or those of the publisher, the editors and the reviewers. Any product that may be evaluated in this article, or claim that may be made by its manufacturer, is not guaranteed or endorsed by the publisher.
